# Supplementary material for: Isolation and Functional Analysis of VvWRKY28, a Vitis vinifera WRKY Transcription Factor Gene, with Functions in Tolerance to Cold and Salt Stress in Transgenic Arabidopsis thaliana
Source: Int J Mol Sci. 2022 Nov 2;23(21):13418. doi: 10.3390/ijms232113418 (PMC9658438; doi:10.3390/ijms232113418)

Table S1. Primers used in this study

Primer Name	Primer sequence (5'→3')	Function
VvWRKY28-F	ATGTCTGATGAGCCAAGAGA	Clone full length of *VvWRKY28*
VvWRKY28-R	TCTCTTGGCTCATCAGACAT	Clone full length of *VvWRKY28*
q-PCR-F	CGTTGAAGGCAATCATAAGC	q-PCR for *MbCBF1*
q-PCR-R	TTAGCCCCTGTGGGTAGAAA	q-PCR for *MbCBF1*
Actin-F	GTTGCCCCTGAAGAACACCC	q-PCR for Actin
Actin-R	GAGATGGCTGGAAGAGGACT	q-PCR for Actin
RAB18-F	GCTTGCGATTTGGGATACAG	Clone full length of *RAB18*
RAB18-R	CCAGACGAACCTTCAGCAGT	Clone full length of *RAB18*
AtCOR15A-F	CAACAGAGGAATCACCAGCGA	Clone full length of *AtCOR15A*
AtCOR15A-R	CTCTGCTGTCTTGTCGTGGTGT	Clone full length of *AtCOR15A*
AtERD10-F	GCAGCAGGAGGAGAAGGG	Clone full length of *AtERD10*
AtERD10-R	CACCAGGAAGAAGCCCATC	Clone full length of *AtERD10*
AtPIF4-R	TGGAAGGCATTCTTGTTG	Clone full length of *At PIF4*
At PIF4-F	GCAGCAGGAGGAGAAGGG	Clone full length of *At PIF4*
AtCOR47-F	TGGTTGTAACGGAGCATC	Clone full length of *AtCOR47*
AtCOR47-R	CCCCAAGAAATCAAACAA	Clone full length of *AtCOR47*
AtICS1-F	CAACGAGGGGAAGATAAAAGTGT	Clone full length of *AtAtICS1*
AtICS1-R	AGCCAGATGATTTTGGAGCCT	Clone full length of *AtAtICS1*
AtNCED3-F	ATGGCTTCTTCACGGCACGG	Clone full length of *AtNCED3*
AtNCED3-R	TTCCTTTGCCCTCGGACG	Clone full length of *AtNCED3*
AtCAT1-F	CGCCATGCCGAAAAATACCC	Clone full length of *AtCAT1*
AtCAT1-R	CTTGCCTGTCTGAATCCCAGGAC	Clone full length of *AtCAT1*
AtP5CS1-F	GATACGGATATGGCAAAGCG	Clone full length of *AtP5CS*

AtP5CS1-R	CCAAGTCCAAATCGGAAACC	Clone full length of *AtP5CS*
AtSnRK2.4-F	GAGGAAATGGGGATGCAGAT	Clone full length of *AtSnRK2.4*
AtSnRK2.4-R	CGAGCCAAAGGACCATACAT	Clone full length of *AtSnRK2.4*
AtCAT2-F	TGCTGCTGGTCTTTCTGCGTTTT	Clone full length of *AtCAT2*
AtCAT2-R	GGAGGGGAACAAACGGGCACA	Clone full length of *AtCAT2*
AtPOD1-F	GTTTTGAACAGCAGTGAGGGTG	Clone full length of *AtSOD2*
AtPOD1-R	GCCTGCGTTTCCAGTAGCCA	Clone full length of *AtSOD2*
AtSOS2-F	AGAAAATGAGAAGAGTGGGCAAGT	Clone full length of *AtSOS2*
AtSOS-R	GTGTTTTGGGATTGGGGTCA	Clone full length of *AtSOS2*
AtP5CS1-F	GAGGAAATGGGGATGCAGAT	Clone full length of *AtP5CS1*
AtP5CS1-R	TTCTCACTTCTCCACTTGCG	Clone full length of *AtP5CS1*

Figure S1. The predicted amino acid sequence and nucleotide of *VvWRKY28* gene

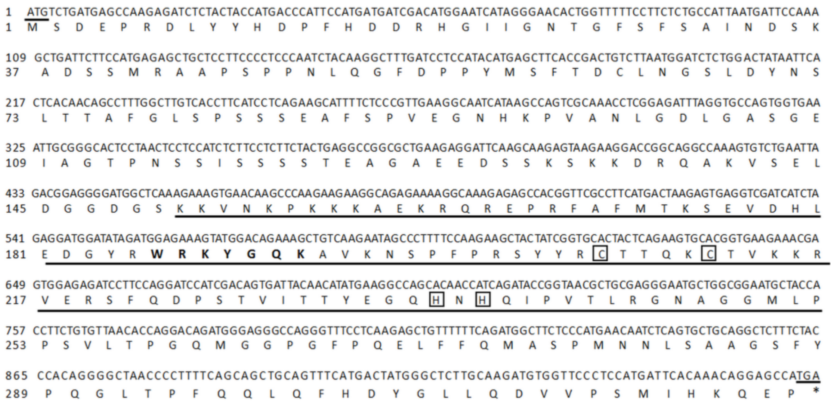

Supplement: Supplementary file 1 [file ijms-23-13418-s001.zip › ijms-1958857-supplementary.pdf]
